# Literary literacy and mental health in contemporary China: evidence from the China family panel studies

**DOI:** 10.3389/fpsyg.2026.1922601

**Published:** 2026-08-31

**Authors:** Hang Shi

**Affiliations:** Department of Basic Courses, Xi’an Railway Vocational & Technical Institute, Xi’an, Shaanxi, China

**Keywords:** China family panel studies, language-cognitive capacity, literary literacy, mental health, reading participation

## Abstract

**Background:**

Mental health problems impose a substantial social and economic burden in China. Yet population-based mental-health research has paid limited attention to literary literacy as a cultural resource embedded in everyday life. This study examines literary literacy as a multidimensional construct associated with adult mental health.

**Methods:**

We analysed 114,448 person-wave observations from six China Family Panel Studies waves (2010, 2014, 2016, 2018, 2020, and 2022). The reconstructed literary-literacy score combines two direct domains—reading participation and language-cognitive capacity—using equal weights after within-wave standardization. Respondent education, parental education and family cultural resources were removed from the score and entered as covariates. The analysis used generalized propensity-score inverse probability weighting, covariate adjustment, individual fixed effects, a generalized additive percentile profile, an observed-variable parallel-path structural equation model and product-term moderation models.

**Results:**

In the weighted covariate-adjusted model, a one-standard-deviation increase in reconstructed literary literacy was associated with a 0.058-SD higher mental-health score (95% CI [0.046, 0.070]). The within-person estimate was smaller (*β* = 0.009, 95% CI [−0.001, 0.019]). In component models, the language-cognitive estimate was positive (*β* = 0.075, 95% CI [0.066, 0.084]), whereas the average reading-participation estimate was close to zero (*β* = −0.003, 95% CI [−0.009, 0.004]). The parallel-path model identified indirect coefficients through language expression, future confidence, interpersonal trust and life satisfaction, all with 95% confidence intervals above zero. The literary-literacy gradient was larger among women, rural residents, lower-income and lower-education groups, and adults aged 60 or older.

**Conclusion:**

Reconstructing literary literacy from direct reading and language-cognitive indicators produced a positive adjusted association with adult mental health. The results locate the strongest average contribution in language-cognitive capacity and show that the joint profile varies across social groups.

## 1 Introduction

Mental health has become a major public-health and social-policy issue in China, with increasing concerns regarding population mental health and social inequalities (World Health Organization China, 2019; Xu et al., 2016). The China Mental Health Survey reported a lifetime prevalence of mental disorders of 16.6% (Huang et al., 2019). More recent evidence indicates substantial symptom burden after the nationwide adjustment of COVID-19 control policies: in a December 2022 online survey, 55.7% of participants reported depressive symptoms and 30.1% reported anxiety symptoms (Qin et al., 2023). Because that study used convenience sampling and symptom-screening instruments, these percentages should not be interpreted as diagnostic prevalence. Together, the national diagnostic estimate and the post-COVID symptom evidence underscore a continuing need to examine the social and cultural conditions associated with mental health.

In this article, literary literacy refers to the conjunction of enacted reading and language-related cognitive resources. Reading participation captures engagement with books, while language-cognitive capacity captures performance on word-recognition or verbal-memory tasks. This definition keeps the construct close to observable literary practice and language use. Education, parental education and family cultural resources are treated as conditions that shape access to literary literacy rather than as parts of the score itself. Cultural-capital theory remains relevant because these direct resources are socially distributed and may become embodied capacities used in everyday life (Bourdieu, 1984). Contemporary work on reading and narrative processing further suggests links with meaning integration, perspective taking and emotional articulation (Lin et al., 2019; Hartung et al., 2021; Kidd and Castano, 2013; Panero et al., 2016; Jensen et al., 2024; Dadswell and Bungay, 2025).

Three gaps motivate the study. First, literary and educational research usually examines literacy as a competence to be cultivated, whereas adult mental health is rarely the outcome (Liu et al., 2023; Pan et al., 2022; Wang et al., 2020). Second, public-health research on depression, stress and social support seldom incorporates reading practice and language-related resources in the same model. Third, studies of reading or cultural engagement often examine one indicator at a time. The present design therefore separates the focal literary-literacy score from its educational and family antecedents, estimates the average association, examines the joint two-domain profile and tests psychosocial and social-group patterning.

The Chinese context is especially informative because educational expansion, rural–urban inequality, uneven cultural access and mental-health burden coexist at large scale. This study asks four questions. First, is the reconstructed literary-literacy score associated with adult mental health after adjustment for education, parental education and family cultural resources? Second, how does mental health vary across the joint profile of reading participation and language-cognitive capacity? Third, do language-expression capacity, future confidence, interpersonal trust and life satisfaction carry parallel indirect associations? Fourth, does the literary-literacy gradient differ by gender, household registration, income, education and age?

## 2 Methods

### 2.1 Data

We used six waves of the China Family Panel Studies (CFPS): 2010, 2014, 2016, 2018, 2020, and 2022 (China Family Panel Studies, 2024). The nationally representative CFPS uses multistage probability sampling and its 2010 baseline covered 25 provincial-level units (Xie and Hu, 2014). The 2012 wave was not included in the primary analysis because it did not contain a reading-participation item comparable with the other waves. We restricted the sample to adults aged 18–90 years; observations above age 90 were sparse and had substantial missingness in the focal measures. The analytic file contained 114,448 person-wave observations from 45,481 individuals, averaging 2.52 waves per person. The individual fixed-effects sample contained 100,646 observations from 31,679 individuals with at least two eligible waves. Wave-specific sample sizes are reported in Table 1.

**Table 1:** Wave-specific measurement logic for literary literacy and mental health.

Wave	Reading participation	Language-cognitive capacity	Mental health	Primary-model role
2010	Leisure-reading frequency	34-item word recognition	K6	Included; *n* = 32,143
2012	No comparable item	Immediate verbal recall	CES-D-20	Excluded from primary models
2014	Books read in past year	34-item word recognition	K6	Included; *n* = 30,470
2016	Books read in past year	Immediate verbal recall	CES-D-20	Included; *n* = 25,756
2018	Books read in past year	34-item word recognition	CES-D-8	Included; *n* = 23,051
2020	Books read in past year	Immediate verbal recall; rotating module	CES-D-8	Included; *n* = 2,320
2022	Books read in past year	34-item word recognition; rotating module	CES-D-8	Included; *n* = 708

Reading and language-cognitive measures were standardized within wave before equal-weight index construction. The 2012 wave was excluded from the primary analysis because no comparable reading-participation item was available. The rotating cognitive modules account for the smaller 2020 and 2022 samples.

### 2.2 Measurement

#### 2.2.1 Dependent variable

Mental health was measured with the six-item Kessler distress scale in 2010 and 2014, the CES-D-20 in 2016, and the CES-D-8 in 2018, 2020, and 2022. Items were oriented so that higher scores represented better mental health and then standardized within wave. This places respondents relative to others assessed with the same instrument in the same survey year. For Table 2, mental-health status was defined using established cut-offs: K6 distress scores of 13 or higher, CES-D-20 scores of 24 or higher and CES-D-8 scores of 15 or higher were classified as mentally unhealthy (Radloff, 1977; Turvey et al., 1999; Lee et al., 2012). Instrument-specific models were estimated for the K6, CES-D-20 and CES-D-8 subsets.

**Table 2:** Descriptive characteristics of the analytic sample.

Variable	Full sample	Mentally healthy	Mentally unhealthy	*p* value
Mentally healthy, *n* (%)	79,371 (69.4)	79,371 (100)	0 (0)	–
Mental-health score (*z*)	0.00 (1.00)	0.27 (0.74)	−0.61 (1.22)	<0.001
Literary-literacy index (*z*)	−0.00 (1.00)	0.04 (1.00)	−0.09 (0.98)	<0.001
Reading participation (*z*)	−0.00 (1.00)	0.02 (1.02)	−0.05 (0.95)	<0.001
Language-cognitive capacity (*z*)	−0.00 (1.00)	0.04 (0.99)	−0.10 (1.02)	<0.001
Age (years)	47.86 (16.13)	47.06 (16.11)	49.67 (16.03)	<0.001
Education (years)	7.33 (4.72)	7.48 (4.72)	7.00 (4.71)	<0.001
Log individual income	6.80 (4.28)	6.19 (4.59)	8.20 (3.04)	<0.001
Parental education (years)	3.23 (3.49)	3.32 (3.53)	3.03 (3.38)	<0.001
Family cultural resources (*z*)	0.05 (0.89)	0.07 (0.91)	−0.01 (0.85)	<0.001
Women, *n* (%)	59,035 (51.6)	39,703 (50.0)	19,332 (55.1)	<0.001
Rural household registration, *n* (%)	82,677 (72.2)	55,996 (70.5)	26,681 (76.1)	<0.001

The analytic sample included 114,448 adult person-wave observations from six CFPS waves. Continuous variables are mean (SD); categorical variables are *n* (%). Mental health, literary literacy and its two components are shown as standardized scores. *p* values compare the mentally healthy and mentally unhealthy groups using *t* tests or chi-square tests.

#### 2.2.2 Literary-literacy variables

Literary literacy was reconstructed from two direct domains. Reading participation was measured by leisure-reading frequency in 2010 and the number of non-work, non-examination books read during the previous 12 months from 2014 onward; book counts were transformed as log(1 + books). Language-cognitive capacity was measured with the comparable word-recognition score in 2010, 2014, 2018, and 2022 and with immediate verbal recall in 2016 and 2020. The word-recognition module contains 34 progressively difficult items and the score records the respondent’s attained item level; the verbal recall score combines the two immediate recall trials. Each domain was standardized within wave, the two *z* scores were averaged with equal weights, and the resulting score was re-standardized within wave. Respondent education, parental education and household cultural-resource indicators were explicitly excluded from this primary score. The 2020 and 2022 cognitive tests were administered through rotating modules, which explains their smaller eligible samples. Table 1 records the complete wave logic.

#### 2.2.3 Covariates

The adjustment set included age and age squared, gender, rural or urban household registration, respondent education, log individual income, parental education, family cultural resources, survey wave and province. Parental education was calculated from the available maternal and paternal education reports. Family cultural resources combined within-wave standardized household book holdings and cultural-education expenditure. Missing continuous covariates were replaced with the wave-specific median and accompanied by missing-value indicators.

This specification responds directly to the distinction between the focal construct and its antecedents. Education, parental education and household cultural resources now enter the regression models only as adjustment variables. Their coefficients are not used to calculate literary literacy.

Survey-wave indicators address common period changes, while province indicators capture broad regional differences. Income and education were also dichotomized at the within-wave median for moderation analyses. Older age was defined as 60 years or above. Descriptive characteristics are presented in Table 2.

### 2.3 Statistical analyses

We first compared characteristics between the mentally healthy and mentally unhealthy groups using *t* tests for continuous variables and chi-square tests for categorical variables. The main sequence comprised a crude model, a generalized propensity-score inverse probability weighted model, a weighted model with covariate adjustment and an individual fixed-effects model. A generalized additive model estimated the fitted literary-literacy profile from the 25th through 90th percentiles. An observed-variable parallel-path structural equation model estimated the four indirect paths, and five separate product-term models tested moderation.

The stabilized generalized propensity score was estimated for the continuous literary-literacy score from the observed adjustment variables. Weights were trimmed at the first and 99th percentiles. The weighted outcome model included the full covariate set, wave indicators and province indicators. The individual fixed-effects model demeaned mental health, literary literacy, age, income and family cultural resources within person and retained survey-wave indicators. Standard errors were clustered by individual throughout.

Because literary literacy was measured as a continuous standardized index, inverse probability weights were constructed under a generalized propensity-score framework. Let LLV_i_ denote the literary-literacy index for observation i, and let X_i_ denote the vector of observed covariates. The stabilized weight was defined as follows:

$$wi_{=\frac{f(\text{LLVi})}{f(\text{LLVi}|\mathrm{Xi})}}$$

In this expression, f(LLV_i_) is the estimated marginal density of literary literacy, and f(LLV_i_|X_i_) is the estimated conditional density of literary literacy given observed covariates. In implementation, the conditional density was estimated by modeling LLV_i_ as a function of observed covariates and assuming normally distributed residuals. The weighted outcome model was specified as follows:

$$Y_{i}=\alpha +\mathrm{\beta LL}V_{i}+\gamma X_{i}+\lambda_{t}+\mu_{\rho }+\varepsilon_{i}$$

In this model, Yi denotes standardized mental health, LLVi denotes the reconstructed literary-literacy score, Xi denotes the covariate vector, *λt* denotes survey-wave indicators, μp denotes province indicators and εi denotes the error term. The coefficient *β* gives the adjusted change in standardized mental health associated with a one-standard-deviation difference in literary literacy.

The fitted literary-literacy profile was estimated with a smooth term for the reconstructed standardized score in a generalized additive model. The same demographic, socioeconomic, family, wave and province terms used in the adjusted model were included. Predictions were generated from the 25th through 90th percentiles and expressed as differences from the 25th-percentile reference. Separate adjusted curves were fitted for household registration, gender and income groups.

The profile was displayed as a continuous curve with a 95% confidence band. Values at the 25th, 50th, 75th, and 90th percentiles were marked to make the magnitude and shape directly readable. The three lower panels use the same percentile scale and outcome metric for the rural–urban, women–men and lower–higher income comparisons.

The parallel-path structural equation model used the 2014 and 2016 waves, where all four pathway variables were available. Literary literacy was the focal predictor; interviewer-rated language-expression capacity, future confidence, interpersonal trust and life satisfaction were entered simultaneously; and standardized mental health was the outcome. Each pathway variable and mental health was regressed on literary literacy and the adjustment variables. Residual covariances among the four pathway variables were freely estimated. The observed-variable model was just identified. Indirect coefficients and 95% confidence intervals were calculated from the product of the corresponding a and b paths using cluster-robust maximum likelihood estimates.

Moderation was tested in five separate adjusted models by adding the product of literary literacy with female status, rural registration, lower income, lower education or age 60 and above. The product-term coefficient and its 95% confidence interval provide the formal comparison between groups; group-specific slopes were calculated to aid interpretation.

Analyses were conducted in R 4.4.2. Linear models used cluster-robust sandwich standard errors; the joint surface used mgcv; and the parallel-path structural equation model used lavaan with robust maximum likelihood and individual-level clustering.

## 3 Results

### 3.1 Adjusted association between literary literacy and mental health

**Figure 1:**
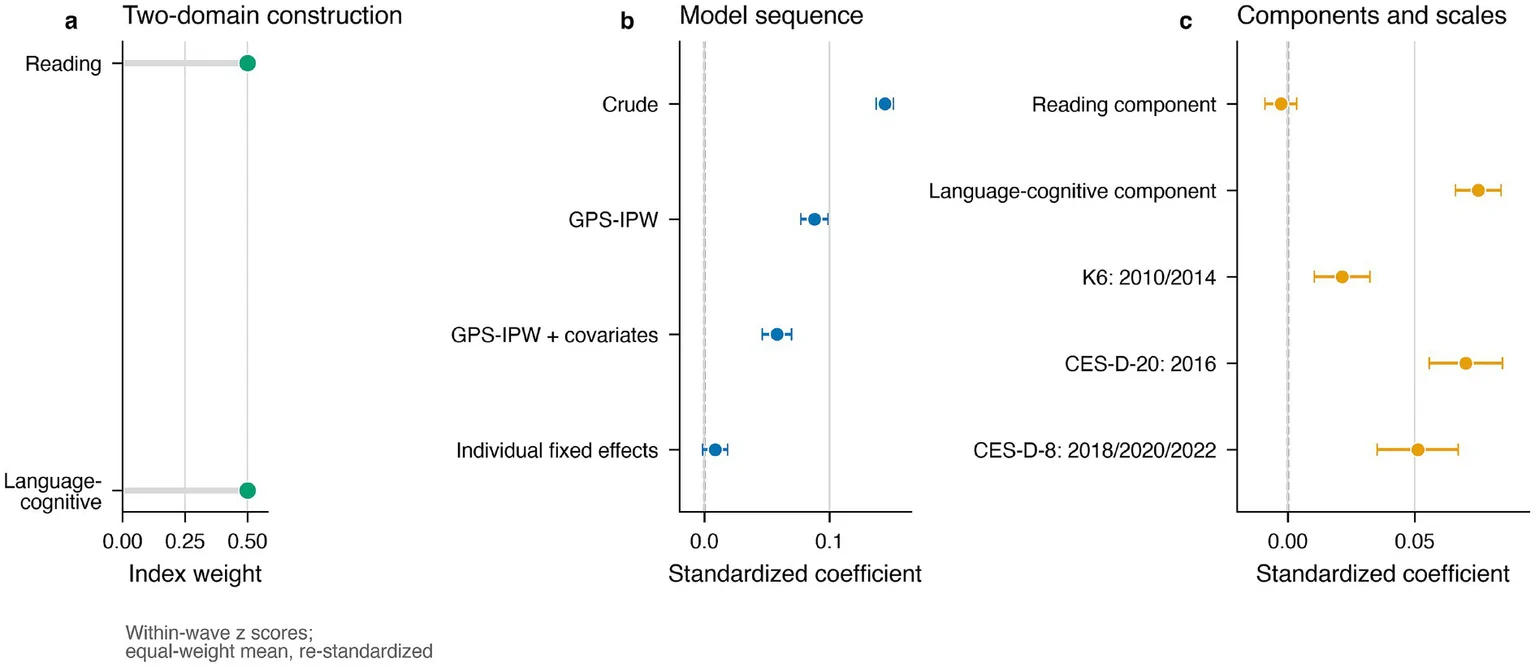
Construction and fitted estimates for the reconstructed literary-literacy score. Panel **(a)** shows the equal-weight construction from reading participation and language-cognitive capacity. Panel **(b)** presents crude, generalized propensity-score weighted, weighted covariate-adjusted and individual fixed-effects estimates. Panel **(c)** reports component-specific and instrument-specific estimates. Points are standardized coefficients; horizontal intervals are 95% confidence intervals.

Figure 1b shows the model sequence. The crude coefficient was 0.144 (95% CI [0.137, 0.151]). It decreased to 0.088 after generalized propensity-score weighting (95% CI [0.077, 0.099]) and to 0.058 after weighting plus covariate adjustment (95% CI [0.046, 0.070]). The unweighted adjusted estimate was 0.041 (95% CI [0.032, 0.049]). In the within-person fixed-effects model, the estimate was 0.009 (95% CI [−0.001, 0.019], *p* = 0.089). Instrument-specific coefficients were 0.021 for K6 (95% CI [0.010, 0.032]), 0.070 for CES-D-20 (95% CI [0.056, 0.085]) and 0.051 for CES-D-8 (95% CI [0.035, 0.067]).

All five product terms were positive. The interaction coefficient was 0.042 for women (95% CI [0.028, 0.055]), 0.066 for rural registration (95% CI [0.052, 0.081]), 0.100 for lower income (95% CI [0.087, 0.112]), 0.087 for lower education (95% CI [0.071, 0.102]) and 0.147 for age 60 or above (95% CI [0.130, 0.164]). The corresponding literary-literacy slopes were 0.063 among women, 0.065 among rural residents, 0.070 in the lower-income group, 0.074 in the lower-education group and 0.155 among older adults (Table 3).

**Table 3:** Main, fixed-effects and scale-specific associations between literary literacy and mental health.

Analysis	*N* person-waves	*N* individuals	*β*	SE	95% CI
Crude	114,448	45,481	0.144	0.003	[0.137, 0.151]
GPS-IPW	114,448	45,481	0.088	0.006	[0.077, 0.099]
GPS-IPW + covariates	114,448	45,481	0.058	0.006	[0.046, 0.070]
Unweighted + covariates	114,448	45,481	0.041	0.004	[0.032, 0.049]
Individual fixed effects	100,646	31,679	0.009	0.005	[−0.001, 0.019]
Reading participation	114,448	45,481	−0.003	0.003	[−0.009, 0.004]
Language-cognitive capacity	114,448	45,481	0.075	0.005	[0.066, 0.084]
K6: 2010/2014	62,613	40,405	0.021	0.006	[0.010, 0.032]
CES-D-20: 2016	25,756	25,756	0.070	0.007	[0.056, 0.085]
CES-D-8: 2018/2020/2022	26,079	23,781	0.051	0.008	[0.035, 0.067]

Coefficients are standardized estimates with individual-clustered 95% confidence intervals. GPS-IPW weights were trimmed at the first and 99th percentiles. The fixed-effects model used respondents with at least two eligible observations. Component and instrument-specific rows are separate adjusted models based on the reconstructed two-domain definition.

Figure 1a records the revised two-domain construction and Figure 1c compares the direct components. In separate adjusted models, reading participation showed little average association with mental health (*β* = −0.003, 95% CI [−0.009, 0.004]), whereas language-cognitive capacity had a positive coefficient (*β* = 0.075, 95% CI [0.066, 0.084]). These estimates clarify the relative contribution of the two observed domains without reintroducing education or family background into the literary-literacy score.

### 3.2 Fitted literary-literacy percentile profile

**Figure 2:**
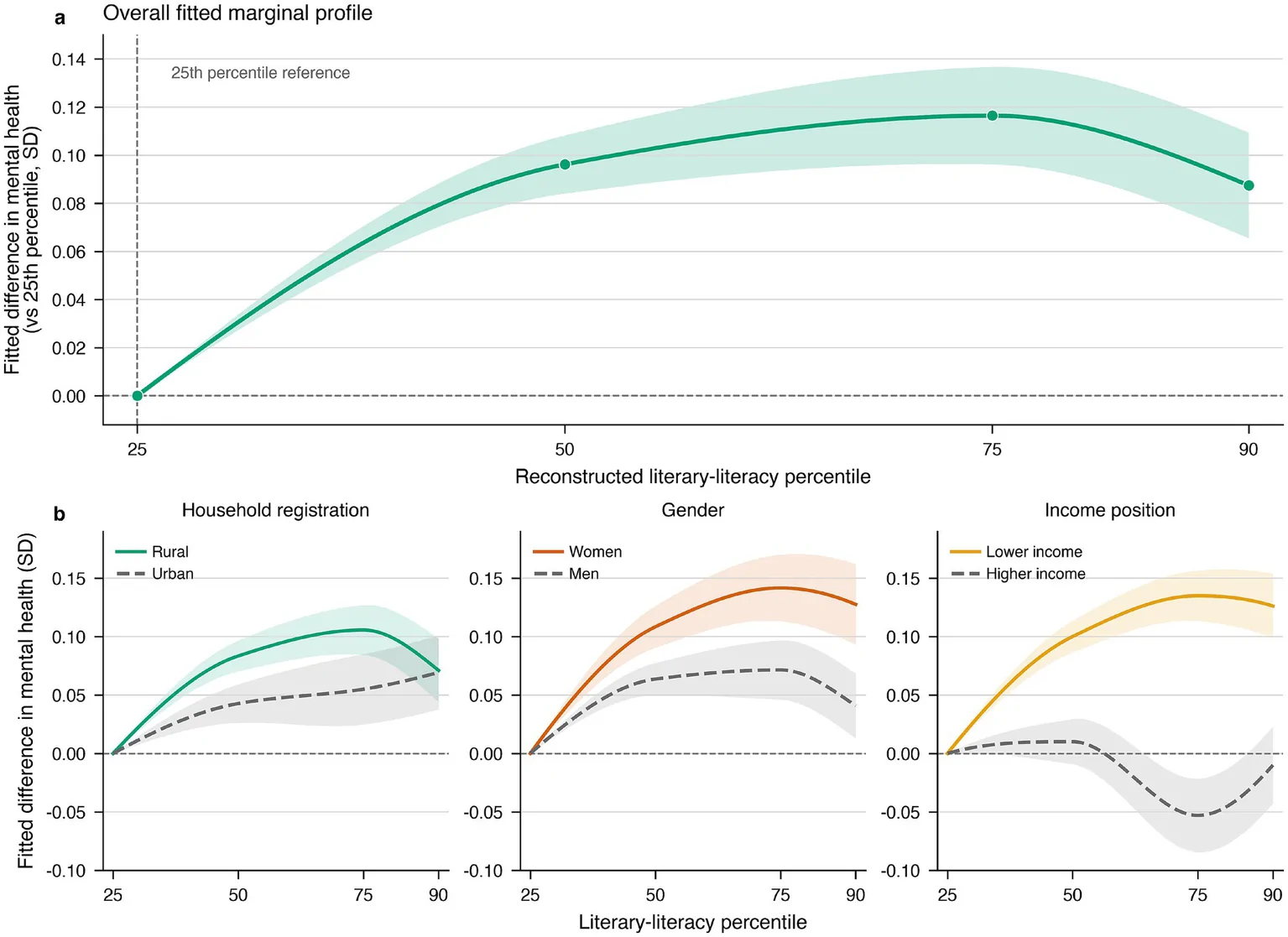
Fitted marginal profile of the reconstructed literary-literacy score. Panel **(a)** shows the overall fitted difference in standardized mental health from the 25th through 90th percentiles, using the 25th percentile as the reference. The shaded area is the 95% confidence interval. Panel **(b)** shows separately fitted curves by household registration, gender and income position, with the same reference and outcome scale.

Figure 2 presents the fitted percentile profile of the reconstructed score. Relative to the 25th percentile, the fitted mental-health difference was 0.096 SD at the 50th percentile (95% CI [0.084, 0.108]), 0.116 SD at the 75th percentile (95% CI [0.096, 0.137]) and 0.087 SD at the 90th percentile (95% CI [0.066, 0.109]). The overall curve rose most rapidly between the 25th and 50th percentiles, reached a broad plateau around the upper-middle range and declined modestly beyond the 75th percentile. The lower panels show larger fitted profiles among rural respondents, women and the lower-income group.

### 3.3 Potential psychosocial pathways and moderation patterns

**Figure 3:**
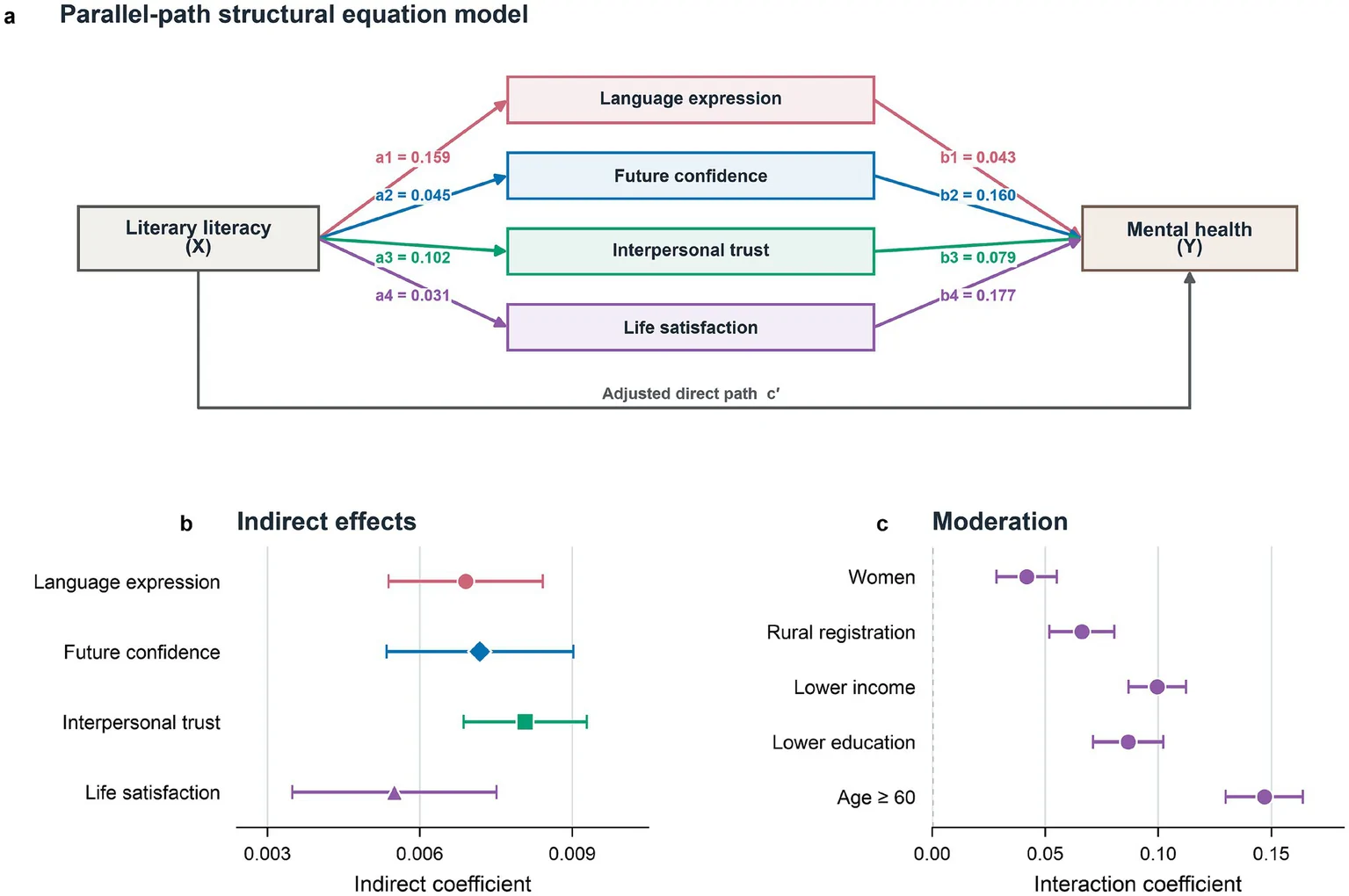
Parallel-path structural equation and moderation estimates. Panel **(a)** reports *a* and *b* path coefficients. Panel **(b)** reports the four indirect coefficients. Panel **(c)** reports interaction coefficients for female status, rural registration, lower income, lower education, and age 60 or above. All intervals are 95% confidence intervals.

Figure 3 presents the parallel-path and moderation results. Literary literacy was positively related to language-expression capacity (*a* = 0.159, 95% CI [0.149, 0.169]), future confidence (*a* = 0.045, 95% CI [0.034, 0.056]), interpersonal trust (*a* = 0.102, 95% CI [0.091, 0.113]) and life satisfaction (*a* = 0.031, 95% CI [0.020, 0.042]). In the simultaneous outcome equation, the corresponding b paths were 0.043 (95% CI [0.034, 0.052]), 0.160 (95% CI [0.149, 0.171]), 0.079 (95% CI [0.071, 0.087]) and 0.177 (95% CI [0.166, 0.188]).

The indirect coefficients were 0.0069 through language expression (95% CI [0.0054, 0.0084]), 0.0072 through future confidence (95% CI [0.0053, 0.0090]), 0.0081 through interpersonal trust (95% CI [0.0069, 0.0093]) and 0.0055 through life satisfaction (95% CI [0.0035, 0.0075]). Interpersonal trust produced the largest estimated indirect coefficient, followed by future confidence and language expression.

The moderation results in Figure 3c place the largest contrast among adults aged 60 or above, followed by the lower-income and lower-education groups. Women and rural-registration respondents also had larger slopes than their comparison groups. Table 4 reports all four indirect paths and all five formal interaction coefficients.

**Table 4:** Structural equation indirect associations and moderation estimates.

Analysis	Target	*a* path [95% CI]	*b* path [95% CI]	Indirect/interaction *β* [95% CI]
Parallel path	Language-expression capacity	0.159 [0.149, 0.169]	0.043 [0.034, 0.052]	0.0069 [0.0054, 0.0084]
Parallel path	Future confidence	0.045 [0.034, 0.056]	0.160 [0.149, 0.171]	0.0072 [0.0053, 0.0090]
Parallel path	Interpersonal trust	0.102 [0.091, 0.113]	0.079 [0.071, 0.087]	0.0081 [0.0069, 0.0093]
Parallel path	Life satisfaction	0.031 [0.020, 0.042]	0.177 [0.166, 0.188]	0.0055 [0.0035, 0.0075]
Moderator	Female status	–	–	0.042 [0.028, 0.055]
Moderator	Rural registration	–	–	0.066 [0.052, 0.081]
Moderator	Lower income	–	–	0.100 [0.087, 0.112]
Moderator	Lower education	–	–	0.087 [0.071, 0.102]
Moderator	Age 60 or above	–	–	0.147 [0.130, 0.164]

The four pathway variables were entered simultaneously in a just-identified observed-variable parallel-path structural equation model. Confidence intervals use robust individual-clustered standard errors. Moderation rows report product-term coefficients from separate adjusted models.

## 4 Discussion

### 4.1 Literary literacy as a cultural resource for mental health

The reconstructed literary-literacy score was positively associated with mental health after education, parental education and family cultural resources were moved into the adjustment set. The weighted adjusted coefficient was 0.058, about two-fifths of the crude coefficient. This decline shows that educational and family background account for a substantial part of the observed gradient, while the direct two-domain score retains an independent statistical contribution.

The component results sharpen the interpretation. Language-cognitive capacity carried the clearer average association, while reading participation alone was close to zero in the adjusted linear model. Literary literacy in this study is therefore best understood as a joint profile: reading identifies enacted engagement, and language-cognitive performance indicates the resources available for processing and retaining text.

### 4.2 The joint profile of reading participation and language-cognitive capacity

The percentile profile adds information that the single linear coefficient does not show. The steepest increase appears below the median, followed by a broad upper-middle plateau and a modest decline at the highest percentiles. This shape is consistent with a score that combines a concentrated reading distribution with a more continuously distributed language-cognitive domain.

This pattern accords with work that treats reading as a repeated cultural practice whose meaning depends on accumulated language resources and social context (Fancourt and Finn, 2019; Mak et al., 2024). It also explains why the reconstructed score retains two domains instead of reducing literary literacy to book counts alone.

### 4.3 Parallel psychosocial pathways

The parallel-path model identified indirect associations through language expression, future confidence, interpersonal trust and life satisfaction, suggesting that literary literacy may influence mental health through multiple psychological and well-being pathways (Gross, 1998, 2015; Ryff, 1989).

Contemporary neurocognitive research provides a useful basis for the language-expression route (Gross, 1998, 2015; Ryff, 1989). Reading Chinese narrative fiction engages systems involved in coherent narrative representation and social–emotional processing (Lin et al., 2019). Literary style and narrative emotional intensity also recruit distinguishable semantic-integration and affective systems (Hartung et al., 2021). These findings help locate language expression and meaning integration within the broader reading process.

Life satisfaction is conceptually closer to mental health than the other pathway variables, so its coefficient partly reflects overlap between evaluative wellbeing and psychological symptoms. The four variables were entered in parallel and their residual covariances were estimated, allowing each indirect coefficient to be read net of the other three pathways.

### 4.4 Socially patterned heterogeneity and compensatory cultural resources

The strongest moderation appeared among older adults, lower-income respondents and lower-education respondents, with additional differences by gender and rural registration. A compensatory-resource interpretation is plausible: language and reading resources may be especially salient where material, institutional or social supports are more constrained.

A compensatory-resource interpretation helps make sense of this finding. For groups facing stronger structural constraints, literary literacy may serve as a relatively scarce resource for organizing emotion, interpreting difficulty and accessing symbolic forms of self-support (Cohen and Wills, 1985). This does not mean that culture can substitute for income, health services, social protection or care provision. Rather, it suggests that cultural resources may matter more when other resources are limited. In this sense, mental-health inequality is not only about income, education, medical access and family conditions; it also concerns unequal access to symbolic, linguistic and interpretive resources.

This interpretation is compatible with evidence that cultural engagement and arts-based resources are unevenly distributed, and that their association with mental health varies by social position (Fancourt and Tymoszuk, 2019; Mak et al., 2024). The Chinese context makes this point particularly relevant because educational expansion, rural–urban inequality, uneven cultural access and mental-health burden coexist at large scale. Literary literacy may therefore be one way to examine how cultural resources are implicated in the social production of mental-health differences. These findings are also consistent with the stress-buffering perspective, which emphasizes the protective role of psychosocial resources and social support in maintaining mental health (Cohen and Wills, 1985).

### 4.5 Strengths and implications

This study makes four contributions. It reconstructs literary literacy from direct indicators and separates the score from educational and family antecedents. It reports the six-wave sample structure and the rotating cognitive modules in full. It combines weighted, within-person, nonlinear, structural-equation and moderation analyses around one two-domain definition. Finally, it shows where the average gradient is concentrated and which social groups display the largest differences.

The practical implications follow the revised measurement. Community libraries, reading rooms, digital reading access and adult language-learning programs can support the two actionable domains: opportunities to read and resources for understanding and retaining text. The weak average coefficient for reading participation alone suggests that access initiatives should be paired with activities that build language confidence, comprehension and social engagement. Rural communities, lower-income neighborhoods and older adults are priority settings because their fitted gradients were largest.

### 4.6 Limitations and future research

Several limitations remain. The 2012 wave could not contribute to the primary score because it lacked a comparable reading item. The 2020 and 2022 cognitive tests were rotating modules, which reduced the eligible samples in those years. Reading frequency in 2010 differs from the later annual book-count measure, and word recognition is not identical to verbal recall. Within-wave standardization improves comparability but yields a relative score. The CFPS items do not cover literary interpretation, aesthetic judgment or genre. Finally, the pathway model was limited to 2014 and 2016, when all four pathway variables were jointly observed.

Future studies can improve the measurement by adding direct tests of reading comprehension, textual interpretation and literary judgment, together with more detailed records of reading frequency, duration and genre. Repeated measurement of the same cognitive task would also make within-person change easier to distinguish from module rotation. Intervention studies in libraries, adult education and community reading programs could then test which combinations of reading access and language support produce the most durable mental-health gains.

## 5 Conclusion

A literary-literacy score reconstructed from reading participation and language-cognitive capacity was positively associated with adult mental health after adjustment for education, parental education and family cultural resources (*β* = 0.058, 95% CI [0.046, 0.070]). Language-cognitive capacity showed the clearer average component association, while the percentile profile identified a steep lower-range increase and an upper-middle plateau. Interpersonal trust, future confidence, language expression and life satisfaction formed four parallel indirect paths, and the gradient was largest among older, lower-income and lower-education adults.

## Data Availability

The raw data supporting the conclusions of this article will be made available by the authors, without undue reservation.
